# Transcatheter Tricuspid Valve Replacement: A Feasible Solution to a Real-world Problem

**DOI:** 10.31083/j.rcm2305163

**Published:** 2022-05-05

**Authors:** Sanjana Nagraj, Ythan H. Goldberg, Andrea Scotti, Edwin Ho, Manaf Assafin, Mei Chau, Azeem Latib

**Affiliations:** ^1^Department of Internal Medicine, Jacobi Medical Center/Albert Einstein College of Medicine, The Bronx, NY 14061, USA; ^2^Montefiore-Einstein Center for Heart and Vascular Care, Montefiore Medical Center, Albert Einstein College of Medicine, The Bronx, NY 10461, USA

**Keywords:** percutaneous tricuspid valve replacement, tricuspid valve, tricuspid regurgitation, transcatheter tricuspid valve interventions

## Abstract

**Background and Objective:**

As one of the most prevalent valvular pathologies affecting millions globally, moderate-to-severe tricuspid regurgitation (TR) predisposes to increased mortality. Despite the well-established risk of adverse outcomes, an overwhelming majority of TR patients are managed conservatively due to challenges associated with timely diagnosis, clinical course of the disease, competing comorbities that carry prohibitive surgical risk, and poor surgical outcomes. These challenges highlight the importance of transcatheter tricuspid valve replacement (TTVR) which has restructured TR management in promising and innovative ways.

**Methods:**

We start with an overview of the pathophysiology of TR considering its implications in management. We then elaborate on the current state of TR management, including its limitations, thereby highlighting the unique role of TTVR. This is followed by a review of perioperative considerations such as careful patient selection, role of multimodality imaging, the various imaging techniques that are available and their contribution towards successful TTVR. We then review the valves that are currently available and under investigation, including the latest data available on device efficacy and safety, and highlight the ongoing clinical trials.

**Results and Conclusions:**

TTVR is evolving at an exponential pace and has made its mark in the treatment of severe symptomatic tricuspid regurgitation. The promising results sustained by currently available devices and ongoing investigation of valves under development continue to pave the path for further innovation in transcatheter interventions. However, it is important to acknowledge and appreciate the novelty of this approach, the lack of long-term data on safety, efficacy, morbidity, and mortality, and use the lessons learned from real-world experiences to provide a definitive and reproducible solution for patients with symptomatic TR.

## 1. Introduction

Tricuspid regurgitation (TR) is one of the most common valvular pathologies, occurring in 65–85% of the population [1, 2]. Increasing severity of TR is a well-established independent predictor of increased long-term mortality and poor outcomes in patients with heart failure and reduced ejection (5-year survival 34 ± 4% for severe TR vs. 68 ± 1% for trivial TR) [3]. Despite a significant risk of mortality from TR and an estimated prevalence of 1.6 million in the United States (US), fewer than 8000 tricuspid valve (TV) operations are performed annually [4]. Concordant with current guidelines, an overwhelming share of these interventions are deferred until a left-sided valve surgery is required and only about 20% are isolated TV procedures [5].

In the Framingham Offspring Study conducted more than two decades ago, the prevalence of TR identified by color Doppler echocardiography was >80%, although the severity was reported as trace or mild in most subjects [2]. 1.5% and 5.6% of men and women aged 70–83 years, respectively, had TR that was either moderate or worse in severity [2]. The prevalence of moderate-severe TR in this study population would have been significantly higher had the distribution of study subjects across different age categories paralleled current demographics, in which nearly 20% of the U.S. population is of 65 years of age or older. By 2060, nearly one in four American is projected to be an older adult (estimated 94.7 million people) as per the U.S. Census Bureau report, and a similar trend is anticipated in developed countries worldwide [6]. Considering the current population distribution by age and the anticipated increase in the older adult population, the prevalence of clinically significant TR is bound to increase significantly in coming years.

Unfortunately, the current state of TR management falls short of meeting clinical need and can be explained by several contributing factors. First, there exists an incomplete understanding of the etiology of TR, right ventricular (RV) anatomy and its correlation with RV hemodynamics. Additionally, due to a lack of awareness and/or access to transcatheter interventions, referral for more durable interventions is delayed and the traditional practice of managing TR conservatively continues [1, 7]. As many patients with TR of moderate or worse severity carry a prohibitive surgical risk, they are often assumed incorrectly to be ineligible for transcatheter interventions as well and remain unreferred [1, 7, 8]. In those who undergo surgery, factors that increase the likelihood of poor outcomes include increased cardiopulmonary bypass duration, intraoperative hypothermia, high rates of pacemaker dependency, new dialysis requirements, likelihood of postoperative stroke, and prolonged hospital stay [8]. Among transcatheter tricuspid valve interventions (TTVI), very large TV annular sizes that are beyond the scope of currently available repair devices, unfavorable leaflet morphology and mobility, wide coaptation gaps, presence of transtricuspid pacing leads, and varying direction of regurgitant jets pose difficulties.

## 2. Pathophysiology of Tricuspid Regurgitation

Complex and varied pathophysiologic mechanisms underly TR because of contributing anatomic and hemodynamic factors. Based on the pathophysiological mechanism, TR can be classified as either primary or secondary.

### 2.1 Primary Tricuspid Regurgitation 

In primary TR, an intrinsically defective TV either due to congenital defects or acquired damage exposes an otherwise normal right heart to large volumes. Globally, rheumatic heart disease is the most common etiology of primary TR wherein scarring of valvular leaflets leads to malcoaptation [9]. Other causes of primary TR include Ebstein’s anomaly, carcinoid syndrome, infective endocarditis, iatrogenic causes such as radiation exposure or pacemaker lead implantation, trauma, and myxomatous degeneration [10]. Although primary TR carries an indolent nature and progresses slowly (with acute infective endocarditis the notable exception), the 10-year incidence of developing dyspnea or congestive heart failure is around 57% in asymptomatic patients and mortality is higher in comparison to the general population [11, 12].

### 2.2 Secondary (Functional) Tricuspid Regurgitation

Over 80% of TR cases are functional in etiology, wherein a dilated RV leads to stretching of the TV annulus, tethering of the leaflets, or both [12, 13]. Additionally, right atrial dilatation (e.g., from atrial fibrillation) can independently lead to annular dilatation. Functional TR occurs as four distinct morphological subtypes [13]. Left-heart related TR is the most common subtype, wherein primary left-sided myocardial or valvular disease increase left atrial pressure resulting in pulmonary hypertension and exposure of the RV to a high afterload. RV dilation in response to the high afterload leads to passive stretching of the TV annulus, tethering of the leaflets, inadequate leaflet coaptation, and ultimately TR [13]. In right-ventricular disease related TR subtype, primary RV disease such as arrhythmogenic right ventricular cardiomyopathy or inferior infarct result in RV dysfunction, papillary muscle displacement, and subsequent tethering of TV leaflets [13, 14]. The third subtype, precapillary pulmonary hypertension related TR occurs subsequent to high RV afterload from increased pulmonary pressures, and in the absence of an inciting left-sided disease. This leads to apical and lateral displacement of papillary muscles resulting in tethering of valvular leaflets. It is associated with pulmonary arterial hypertension, chronic lung disease and chronic thromboembolic pulmonary hypertension [15, 16]. The isolated subtype of secondary TR is a less frequent entity which occurs independent of left-heart disease, pulmonary hypertension, or RV disease. RV dilation is more prominent in the bases and TV annular dilation follows a pattern distinct from other subtypes, resulting in a planar, and more circular annulus with less leaflet tenting. Additionally, marked right atrial dilation is seen commonly in this condition. Isolated TR has been strongly associated with diastolic dysfunction, atrial fibrillation, and older females with small body surface areas [13, 17, 18, 19]. Survival is dependent on severity, with 10-year survival rate for severe TR being 38% vs.70% for non-severe TR [20].

In both primary and secondary TR, the interaction of RV and TV leads to a vicious cycle, wherein TR begets worse TR. This intricate relationship between TV function and RV hemodynamics, which in later stages progresses independent of the initial inciting left-sided disease explains why RV dysfunction and TR may not always resolve after surgery for left-heart disease [8, 21, 22]. On the contrary, TR carries a sizeable propensity to worsen after left-sided valvular procedures [23]. Worsening severity of TR is independently associated with a progressively increased risk in all-cause mortality, cardiac mortality, and heart failure hospitalizations after adjusting for age, left ventricular ejection fraction (LVEF), RV size and function [3, 24, 25]. Specifically, in functional TR, dilation of the annulus along the anteroposterior commissure results in a planar annulus. Simultaneously, dilation of the annulus laterally stretches the anterior and posterior leaflets along the anteroseptal and posteroseptal commissures, respectively, forming large leaflet gaps. This planar configuration of the annulus plays a central role in the progression of TR, leading to worsening RV dilation, dysfunction, and vice-versa. Additionally, chronic volume and pressure overload induce irreversible RV dysfunction where prognosis is influenced by the severity of concomitant TR.

## 3. Current State of Management of TR

Management of primary TR depends on the severity of regurgitation, RV function, and pulmonary pressures. The ESC/EACTS (European Society of Cardiology/and European Association for Cardiothoracic Surgery) and AHA/ACC guidelines (American Heart Association/American College of Cardiology) make a Class I recommendation of isolated TV surgery for symptomatic severe primary TR. Isolated TV surgery should be considered (Class IIA) for severe primary TR even in the absence of symptoms if concomitant RV dilation or dysfunction is present [26, 27]. These guidelines take into consideration the “clinically silent” nature of TR for a considerable period despite progressive worsening of RV function and the likelihood of developing poor outcomes [12]. For secondary/functional TR, irrespective of symptoms, tricuspid valve surgery is a class I recommendation for severe TR and a class IIA recommendation for mild/moderate TR when left-sided valve surgery is indicated, especially when significant TV annular dilation (≥40 mm) or pulmonary hypertension is present [26, 27]. Contrary to guideline recommendations, current clinical practice tolerates medical management of TR in the absence of another indication for cardiac surgery in the vast majority of cases. However, independent investigators have proposed different algorithms for transcatheter management of TR, wherein the choice of transcatheter intervention can either be guided by the etiology of TR: primary (degenerative) vs. secondary (functional) vs. cardiac implantable electronic device related or based on severeity of TR (moderate vs. severe) if the patient carries prohibitive-surgical risk [28, 29]. In this regard, compared to current guidelines which give a class IIA recommendation of TV intervention in patients with moderate TR undergoing left-sided valve surgery, Russo *et al*. [29] in their TR-severity guided management algorithm, propose reassessment of TR after management of left-heart disease in patients with symptomaticc moderate TR [26, 27, 29].

### 3.1 Surgical Management of TR

Surgical management of TR entails either TV repair or replacement (STVR). TV repair with annuloplasty has been the standard of care surgical treatment as it carries higher overall survival, 76% at 10 years for repair vs. 55% for replacement [27, 30]. Isolated STVR has been identified as a significant independent predictor of postoperative mortality on follow-up (mean duration of follow-up 5.2 ± 4.1 years; HR: 5.1; 95% confidence interval (CI): 2.9–9.1; *p *< 0.0001) [30]. However, long-term survival rates with STVR tend to vary greatly (30–75% at 15-years as reported in different cohorts) [27, 31, 32]. Currently, TV repair with ring annuloplasty is preferred over other TV repair techniques not involving a ring due to lower TR recurrence rates and improved survival as shown in multiple studies [33, 34, 35, 36]. Despite higher survival rates compared to STVR, repair carries considerable mortality. TV annuloplasty is less likely to succeed in patients with significant RV dysfunction and valve tenting as apical displacement of leaflets and sub-valvular apparatus preclude accurate measurements of the TV [7, 8, 21, 22]. While those who undergo surgery have considerable risk of perioperative and long-term mortality, a large proportion of patients with TR have prohibitive-surgical risk due to multiple comorbidities or have distorted valves that are unamenable to surgical repair.

### 3.2 Transcatheter Tricuspid Valve Repair

Transcatheter Tricuspid Valve Repair (TTVr) offers a range of approaches using either coaptation or annuloplasty devices. As nearly 90% of TR in adults is functional, most repair devices aim to improve coaptation directly by way of approximating the leaflets or indirectly by repairing annular dilation (either suture-based or ring-based) [37]. Currently, the majority of available data on TTVr stems from coaptation devices, although short and intermediate-term outcomes have been reported for annuloplasty devices. Transcatheter edge-to-edge repair (TEER) utilizing MitraClip/TriClip (Abbott Park, IL, USA) or PASCAL (Edwards Lifesciences, Irvine, CA, USA) aim to reduce the coaptation gap by replicating the Clover technique [38]. After promising short-term results of the TRILUMINATE early feasibility trial (NCT03227757), recruitment for TRILUMINATE Pivotal trial (NCT03904147) is underway and expected to provide long-term data on efficacy and safety of TriClip compared to optimal medical therapy alone (OMT) [39]. Similarly, the CLASP TR early feasibility study (NCT03745313) showed that the PASCAL transcatheter valve repair system results in sustained TR reduction and improvement in quality of life with a low major adverse event rate during 6-month follow up [40]. To evaluate the long-term efficacy of PASCAL and OMT in comparison with OMT alone, the relatively large-sized CLASP II TR trial (NCT04097145) is underway at multiple centers in the US. Despite these promising early results from TEER, patients with extreme annular dilation and/or wide leaflet gaps, as well as those with suboptimal transesophageal echocardiography (TEE) image quality were excluded from the trials [41].

Suture-based and ring-based annuloplasty devices have their own set of merits and limitations. The TriCinch system, which is suture-based, achieved an 85% procedural success rate in the PREVENT (Transcatheter Treatment of Tricuspid Valve Regurgitation With the TriCinch System) trial (NCT02098200) but late detachment of the anchor, hemopericardium, and risk of injury to the right coronary artery hampered procedural success [42]. An early feasibility study of the ring-based Cardioband tricuspid valve reconstruction system (Edwards Lifesciences, Irvine, CA, USA) demonstrated excellent procedural outcomes and no 30-day mortality [43]. However, use is limited by extreme annular dilation and operator experience considering the high procedural complexity compared to TEER [41].

The pooled outcomes of transcatheter tricuspid valve repair, inclusive of both leaflet-directed and annulus-reshaping repair devices have been evaluated. In a recent meta-analysis of 771 patients with moderate or worse TR who underwent TTVr, significant improvement in functional status (35% with New York Heart Association (NYHA) functional class III or IV compared to 84% at baseline; risk ratio: 0.23; 95% CI: 0.13–0.40; *p *< 0.001) and reduction in TR severeity were noted over a mean follow-up of 212 days [44]. Similarly, in another pooled analysis of 454 patients, wherein at least 95% had severe TR, similar improvements in functional status were observed [45]. However, left- and right ventricular function did not change significantly [45].

### 3.3 Transcatheter Tricuspid Valve Replacement (TTVR)

Symptomatic severe TR despite maximum tolerated medical therapy forms the basis of TTVR in patients who have prohibitive surgical risk and factors preventing successful transcatheter repair as detailed above. Table 1 outlines favorable and unfavorable attributes of currently available transcatheter tricuspid valve interventions with a focus on leaflet- and annulus-directed repair devices, and orthotopic and heterotopic replacement devices. Pre-procedural decision making, including specific anatomic and operative considerations, pertinent imaging modalities, and currently available replacement devices are described in the following sections.

**Table 1.** **Attributes of transcatheter tricuspid valve interventions**.

Intervention	Favorable attributes	Unfavorable attributes
Transcatheter Tricuspid Valve replacement		
Orthotopic Valve implantation	∙ Organic etiology of TR with either rheumatic leaflet thickening, leaflet perforation or shortening, or very large leaflet prolapse	∙ Severe RVD and severe PH
	∙ Coaptation gap >7 mm	∙ Excess tricuspid annular dilation >70 mm
	∙ Potential for complete elimination of TR	∙ Risk of RCA obstruction
	∙ Intrepid Device: recapturable and retrievable	∙ Unfavorable device angle of approach
	∙ LuX valve: Adaptive skirt helps reduce paravalvular leak by conforming to the anatomy of tricuspid annulus	∙ Depending on the device may require large-bore delivery system (Intrepid), transapical approach/mini-thoracotomy (LuX valve), transtrial approach (Navi-GATE)
Heterotopic Valve implantation	∙ Annulus diameter >70 mm beyond the scope of currently available orthotopic devices	∙ Risk of hepatic or azygous vein obstruction
	∙ Coaptation gap >7 mm	∙ Severe PH and increased RA pressures risking fracture of bicaval valved stents
	∙ Severe RVD and PH prohibiting implantation of orthotopic valves	∙ Short distance between cavoatrial junction and hepatic vein
		∙ Limited by very large vena cava diameter
		∙ Requires lifelong therapeutic anticoagulation
Transcatheter Tricuspid Valve Repair		
Leaflet-directed repair	∙ Degenerative TR with confined leaflet prolapse or flail	∙ Rheumatic leaflet thickening, leaflet shortening, or very large leaflet prolapse
	∙ Posteroseptal and anteroseptal jet location	∙ Wide coaptation gaps >8.5 mm beyond the scope of coaptation enhancement devices used with Clip
	∙ Functional TR with small coaptation defect (<7 mm) and good leaflet mobility	∙ Dependent on high-quality echocardiographic visualization of the TV
	∙ TriClip has significant operator experience and outcomes data	∙ Anteroposterior jet location
	∙ Central spacer enables reduction of EROA with PASCAL device	∙ Presence of impinging RV leads
		∙ May not eliminate TR completely
Annulus-reshaping repair	∙ Annular dilation as primary mechanism of TR	∙ Limited by extreme annular dilation
	∙ Central jet location	∙ May not eliminate TR completely
	∙ Early outcomes data have been promising	∙ Less operator experience and outcomes data in comparison to leaflet-directed repair
	∙ Leaflet-independent nature allows leaflet-directed repair if required in the future	∙ Dependent on high-quality echocardiographic visualization of the TV
	∙ Favorable course of the RCA with adequate relative distance to the TV annulus	

TR, tricuspid regurgitation; RVD, right ventricular dysfunction; PH, pulmonary hypertension; RV, right ventricle; RCA, right coronary artery; RA, right atrium; EROA, effective regurgitant orifice area; TV, tricuspid valve.

#### 3.3.1 Patient Selection

In the absence of conditions that definitively preclude effective transcatheter repair, choosing between TTVr and TTVR is up to the operator’s judgement. This is especially relevant considering the large number of novel devices, limited long-term data, center-specific involvement in one or more device trials, heterogeneity in operator experience, and regional variability in device availability.

There may exist specific considerations and principles that guide patient selection for TTVR irrespective of the device. Fibrotic or degenerated valve leaflets, as seen in primary TR from rheumatic heart disease, carcinoid syndrome, or valvular prolapse are generally not candidates for repair because the pathologic leaflets are not amenable to maintaining a durable grasp. The same problem exists when leaflets are severely calcified, especially in the potential landing zone, or are retracted creating an unfavorable angle for coaptation devices to securely grasp both leaflets [44]. In these situations, TTVR may be the only transcatheter option. Encountered primarily in patients with secondary TR, severely dilated TV annuli and/or leaflet tethering with large coaptation gaps are unlikely to achieve satisfactory elimination of TR with edge-to-edge repair [46]. Specifically, coaptation gaps >7 mm, effective regurgitant orifice area >1.5 ${\mathrm{cm}}^{2}$, and pacemaker or implantable cardioverter defibrillator (ICD) leads that traverse the tricuspid valve, and restricted leaflet mobility make grasping of the leaflets during TEER challenging [47]. Extreme RV dysfunction with severe pulmonary hypertension is an important consideration, as with near-complete or complete resolution of TR with TTVR there exists a risk of exposing the already-failed RV to high afterload in the postoperative period. Therefore, upon resolution of TR, the depressed RV may fail to exercise an effective systolic ejection sufficient to overcome the high pulmonary vascular resistance potentially worsening RV failure [5].

Pacemaker or implantable cardioverter defibrillator (ICD) leads that traverse the tricuspid valve present a unique set of problems by interfering with leaflet mobility or with leaflet grasping during TEER [48]. Of note, long term data are lacking and caution should be exercised while deploying and seating the prosthetic valve to reduce the likelihood of lead fracture in the future. Another concern with TEER is jet origins that are not central or anteroseptal, as they are associated with a higher likelihood of repair failure compared to TTVR [47].

Quality of life, competing comorbidities, current functional status and anticipated functional improvement should be carefully considered when deciding whether to perform TTVR. Those with a life-expectancy <1 year, quality of life not expected to improve with TTVR, or frailty intolerant of any operative stress, are not considered candidates for TTVR, and maximally tolerated medical therapy should be continued [41]. Another important consideration pre-procedurally is long term bleeding risk. Patients at high risk of bleeding who cannot tolerate life-long anticoagulation required by currently available replacement devices would be better served with transcatheter repair [49, 50].

Intraoperatively, strategizing the timing of deployment is essential to avoid incorrect positioning, as the tricuspid valve’s complex three-dimensional skeleton changes throughout the cardiac cycle [12]. The size, shape, and anchoring mechanism are all potential sources of injury to surrounding structures such as the right coronary artery, AV node, and the bundle of His [51]. Irrespective of the type of TTVI pursued, a fully equipped multidisciplinary heart team is of paramount importance as decisions are guided by device availability, institutional practices, and operator experience.

#### 3.3.2 Role of Imaging

Multimodality imaging plays a central role in the assessment of TV anatomy, severity of regurgitation, right heart function, and concomitant left heart pathology to help guide patient and device selection. Specifically, each device has unique anatomical requirements. Imaging is required most notably for device sizing, but also for ensuring that the delivery system can be positioned properly, anchoring mechanisms can be deployed, RV outflow obstruction will be avoided, and paravalvular leak will be kept to a minimum. Because valve and chamber dimensions can vary significantly with intravascular volume, sizing should be planned close to the date of the procedure while stable volume status is maintained.

3.3.2.1 Transthoracic Echocardiography

Transthoracic echocardiography helps characterize the etiology and severity of TR. It can be used to quantify TV annulus and leaflet parameters, RV function and size, and pulmonary artery pressures, though other modalities may do so more precisely [52]. Quantification of TR by way of quantitative doppler methods, measurement of RV dimensions at the base and mid-cavity, calculation of tricuspid annular plane systolic excursion, and RV free wall strain form a part of the pre-procedural evaluation [52, 53].

3.3.2.2 Transesophageal Echocardiography

Use of TEE involves acquiring multiple views from different depths and plane angles, with simultaneous use of biplane and 3D imaging to fully visualize the TV annulus, leaflets, and the sub-valvular apparatus [54]. TEE plays a crucial role in quantifying TR severity and determining feasibility of TTVR.

3.3.2.3 Computed Tomography

Although echocardiography is the first-line imaging modality for assessing the TV and RV function, their complex anatomy may preclude a complete assessment. As the diameter and shape of the TV annulus change throughout the cardiac cycle, measurements obtained from Computed tomography (CT) can prevent perioperative complications such as prosthesis-annulus mismatch, paravalvular regurgitation and injury to surrounding anatomical structures [55, 56]. CT using a multi-slice scanner system (64-detector row scanner or higher) can obtain a large volume acquisition without compromising temporal or spatial resolution [57]. Retrospectively gated acquisitions are most frequently used during pre-procedural planning of TTVR [57]. Data sets can then be reconstructed in any required plane with the ability to obtain exact measurements at any timepoint in the cardiac cycle.

Necessary information from CT includes annulus size, assessment of RV size and function, co-existing cardiac and pulmonary pathologies, and optimal location for deployment [55, 58]. CT can also identify surrounding structures that may be potential targets of iatrogenic injury such as the right coronary artery and coronary sinus, as well as the position of papillary muscles, moderator band, and trabeculae that may interfere with proper delivery system positioning or device expansion [58, 59]. CT imaging also helps to determine whether the diameter and course of vein access permit device delivery and in defining the fluoroscopic angles that are coplanar with the tricuspid annulus [58].

Specifically for heterotopic valves, CT can obtain accurate measurements of the inferior vena cava (IVC), generally measured during mid-systole at the junction of the IVC and right atrium and at the level of the first hepatic vein. The distance between these two landmarks is also measured to ensure avoiding obstruction of the first hepatic vein [55]. Additional imaging of the right atrium may be required based on the type of caval valve, such as with implantation of the Tricento prosthesis [60].

There are certain considerations to note with CT for preprocedural planning. Measurements are easily influenced by patients’ volume status and measurements obtained pre-procedurally may not match the ones on the day of the procedure. Therefore, careful medical management with adequate diuresis and scanning close to the tentative date of intervention are important in facilitating procedural success [61].

3.3.2.4 Cardiac Magnetic Resonance Imaging

In instances where the severity of TR cannot be confidently determined by echocardiography, cardiac magnetic resonance imaging (MRI) should be considered. As an adjunct to echocardiography and CT, cardiac MRI by way of good temporal and spatial resolution provides detailed anatomic and functional assessment of RV through multiple planes. This is unlike 2D- or 3D-echocardiography which require multiple windows to acquire adequate data. Unlike echocardiography, image quality with MRI is unaffected by patients’ body habitus, lung windows, or breast implants [58, 62]. Additionally, unlike CT angiography, MRI does not involve radiation exposure or use of contrast to assess valvular regurgitation, ventricular volumes, ejection fraction, and myocardial tissue characterization. A disadvantage that is routinely encountered in current clinical practice is incompatibility of both intracardiac and/or non-cardiac implanted devices with MRI.

3.3.2.5 Imaging Summary and Innovations

Overall, CT provides excellent anatomic and quantitative information and is critical for procedural planning. MRI provides useful functional and hemodynamic assessment and can be used to supplement other standard imaging modalities such as echocardiography and CT. Three-dimensional (3D) printing is a relatively new technique where exact replicas of a patient’s cardiac anatomy can be generated based on volumetric imaging data obtained by TEE, CT, and MRI [63]. In addition to enhanced anatomic and hemodynamic understanding, 3D printed models allow for procedural training on patient-specific models. The first-in-human implantation of the NaviGate prosthesis in a patient with a failed tricuspid annuloplasty was guided by procedural simulation on a 3D printed model [64]. 3D printing of the right atrium-inferior vena cava junction has been described to aid heterotopic valve selection by way of fit testing different valve sizes [65]. Use of intracardiac echocardiography with 4D catheters is a promising technique that may replace TEE, thus decreasing the use of general anesthesia in the near future.

## 4. Devices: Updates on Efficacy, Safety, Feasibility

**Fig. 1.**
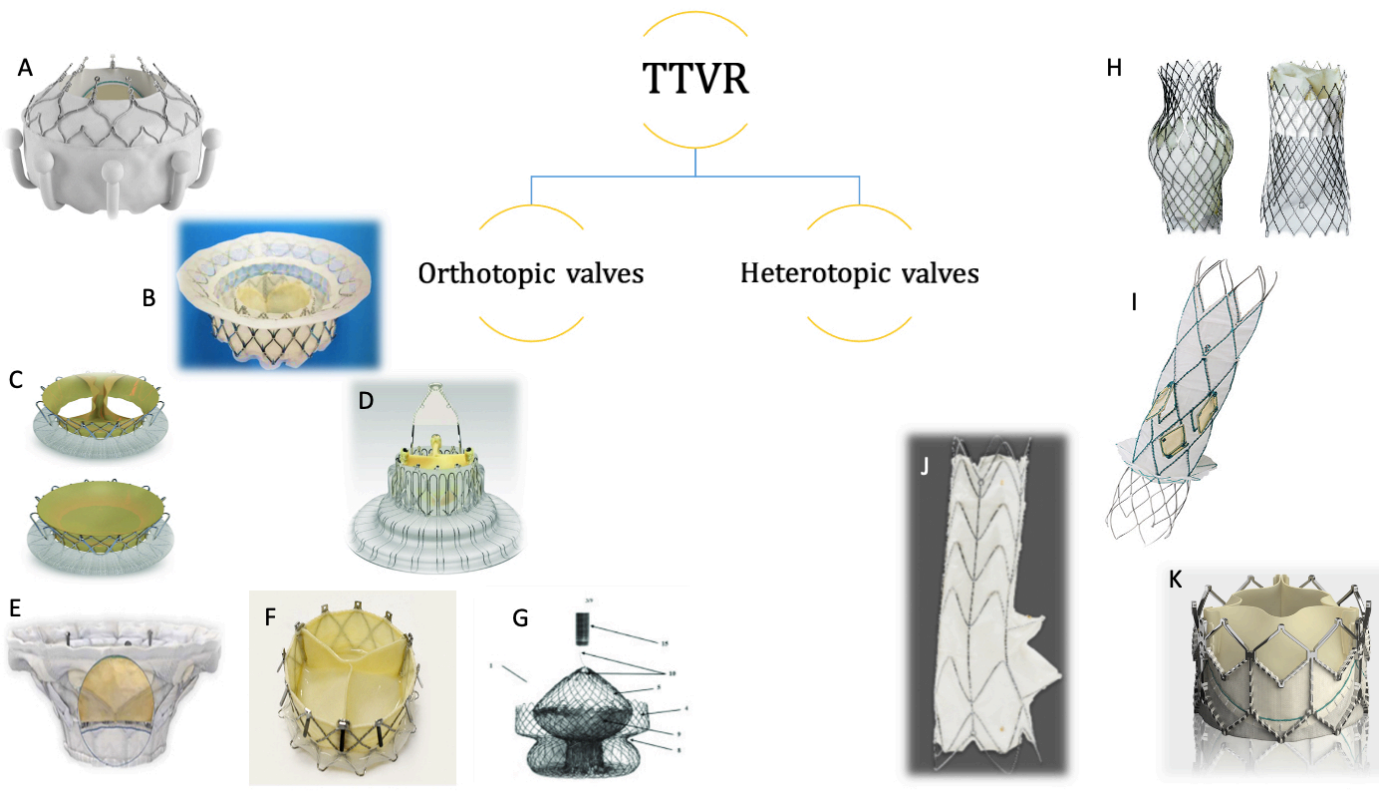
**Orthotopic and heterotopic transcatheter tricuspid valves**. Orthotopic valves (A–G). (A) EVOQUE (Edwards Lifesciences, Irvine, CA, USA). (B) Intrepid (Medtronic Plc, Minneapolis, MN, USA). (C) Trisol (Trisol Medical, Yokneam, Israel). (D) LUX-Valve (Jenscare Biotechnology, Ningbo, China). (E) Cardiovalve (Boston Medical, Shrewsbury, MA, USA). (F) NaviGate (NaviGate Cardiac Structures Inc., Lake Forest, CA, USA). (G) Tricares (TRiCares SAS, Paris, France). Heterotopic valvess (H–K). (H) TricValve (P+F Products + Features, Vienna, Austria). (I) Trillium (Innoventric Ltd, Ness-Ziona, Israel). (J) Tricento (New Valve Technology, Hechingen, Germany). (K) Sapien XT (Edwards Lifescience, Irvine, CA, USA).

TTVR devices can either be orthotopic or heterotopic valves, Fig. 1. Recent developments including device efficacy, safety, and ongoing clinical trials are detailed below and in Table 2.

**Table 2.** **Ongoing clinical trials for currently available transcatheter tricuspid valves**.

Device	Manufacturer	Registered clinical trials	Study design and intervention	Planned enrollment	Primary endpoints	Available results
Orthotopic valves						
EVOQUE	Edwards Lifesciences	TRISCEND (NCT04221490)	Prospective, multi-center, single arm	200	Freedom from device or procedure-related adverse events	∙ 6-month results (56 patients): reduction in TR to none/trace/mild in 100% of patients
						∙ 89% in NYHA Class I/II at 6 months
						∙ 27-point increase in KCCQ over baseline
		TRISCEND II Pivotal Trial (NCT04482062)	Prospective, multi-center, randomized, EVOQUE & OMT vs. OMT alone	775	TR grade reduction and composite endpoint of KCCQ score, NYHA class, and 6MWD	
Intrepid	Medtronic Cardiovascular	TTVR Early Feasibility Study (NCT04433065)	Prospective, multi-center, non-randomized	15	Rate of implant or delivery related SAE	None
TriSol Valve	Trisol Medical	TriSol System EFS Study (NCT04905017)	Prospective, multi-center, non-randomized, first in-human EFS	15	Rate of device-related SAE, technical and procedural success, change in TR from baseline	None
LuX Valve	Jenscare Biotechnology	TRAVEL trial (NCT04436653)	Prospective, multi-center, non-randomized, single arm	150	All-cause death, TR grade reduction ≥2	∙ First-in-human study (12 patients): procedural success with no intraprocedural mortality
Cardiovalve	Boston Medical	Early Feasibility Study of the Cardiovalve System for Tricuspid Regurgitation (NCT04100720)	Prospective, multi-center, non-randomized, single arm	15	Intra-procedural success, technical success, device related SAE	None
NaviGate	NaviGate Cardiac Structures Inc.	None				∙ Compassionate use (35 patients): 30-day mortality: 13.8%
						∙ 100% of patients achieved TR grade ≤2
						∙ EFS approved by FDA in 2019
TRiCares Topaz	TRiCares SAS	None				∙ Compassionate use (2 patients): device success achieved in both cases
Heterotopic valves						
Tric Valve	P + F Products + Features	TRICUS STUDY (NCT03723239)	Prospective, non-randomized, first in-human, single arm EFS	10	MAE at 30 days, change in NYHA class at 6-m	∙ First-in-human experience: successful implantation and improved symptoms at 12-month follow-up
		TRICUS STUDY Euro (NCT04141137)	Prospective, non-randomized, multi-center, single arm	35	MAE and KCCQ score	
Trillium	Innoventric Ltd	Innoventric Trillium Stent Graft First-in-Human Study (NCT04289870)	Prospective, multi-center, non-randomized, single arm, first in-human study	15	Freedom from device or procedure-related SAE, technical success, device success (up to 72 hours), procedural success at 30 days	None
Tricento	New Valve Technology	TRICAR (NCT05064514)	Prospective, single-center, single arm	15	Successful implantation with a 35% reduction in the V-wave pressure in the IVC	∙ First-in-human experience: successful implantation and improved symptoms at 3-month follow-up
Sapien XT and Sapien 3	Edwards Lifesciences	HOVER (NCT02339974)	Prospective, multi-center, non-randomized, single arm	15	Procedural success at 30 days and individual patient success: composite of device success, no re-hospitalizations for RHF or need of mechanical support, and improvement in QOL	∙ Successful caval implantation of the Sapien valve as compassionate use
						∙ TRICAVAL trial terminated prematurely due to a high rate of valve dislodgement

TR, tricuspid regurgitation; NYHA, New York Heart Association; KCCQ, Kansas City Cardiomyopathy Questionnaire; OMT, optimal medical therapy; 6MWD, 6-minute walk distance; EFS, early feasibility study; SAE, serious adverse events; TTVR, transcatheter tricuspid valve replacement; FDA, Food and Drug Administration; MAE, major adverse events; IVC, inferior vena cava; RH, right heart failure; QOL, quality of life.

### 4.1 Orthotopic Valves

#### 4.1.1 EVOQUE System

The EVOQUE system (Edwards Lifesciences, Irvine, CA, USA) consists of bovine pericardial leaflets and an intra-annular sealing skirt with atraumatic anchors that utilize leaflet capture more than radial forces to stabilize device position. It is available in three sizes (44, 48, and 52 mm) and has been designed specifically to accommodate pre-existing leads. Through a transfemoral approach, the 28F delivery system allows for depth control and accurate deployment of the prosthesis with a 93% procedural success rate [66]. Results of the multicenter, first-in-human compassionate use of EVOQUE in 27 patients were promising [66]. 92% of the patients achieved trace or mild TR at one-year post-procedure while all benefited from a reduction in the grade of severity of TR to moderate or less. This was accompanied by significant and persistent functional improvements as 68% of the patients improved to NYHA functional Class II or less over the same period. Notably, the results reflect favorable and continued hemodynamic adaptation to the prosthesis. This is evident as the proportion of patients achieving trace or mild TR improved from 88% at 30 days to 92% at one year with a smaller albeit notable increment in the proportion of patients with NYHA Class II or less (67% at 30 days to 68% at one year). The heart failure (HF) hospitalization rate at 30 days was 0% and 7% between 30 days to one year. This is remarkable as uncorrected moderate to severe TR has a HF hospitalization rate of around 40% and is a known independent predictor of HF readmission [67, 68].

This first-in-human experience was followed by the early feasibility trial TRISCEND (Edwards Transcatheter Tricuspid Valve Replacement: Investigation of Safety and Clinical Efficacy Using a Novel Device; NCT04221490) [69]. Enrolling 200 patients with at least moderate TR into a single-arm, multicenter prospective study, TRISCEND demonstrated high device and procedural success rates. Persistent reduction in TR severity to trace/none or mild at six months occurred in 100% of the patients (improved from 98% at 30 days) with an acceptable composite major adverse event (MAE) rate, comprised mostly of non-fatal bleeding [70]. These results are remarkable as half the study population in TRISCEND had massive or torrential TR, and a 94% procedural success rate was obtained despite a largely elderly population with multiple significant comorbidities (>90% had atrial fibrillation, >90% had ascites, 66% had chronic kidney disease, and nearly 80% had pulmonary hypertension) [71]. Recently published data also reported sustained improvement in quality of life, with nearly 90% of the cohort in NYHA Class I or II at six months [70]. An important concern from TRISCEND was the development or worsening of RV dysfunction. Nearly 20% of the patients developed new moderate-severe RV systolic dysfunction immediately following TTVR, which persisted in 5% of the patients at 30 days [69]. Long-term data on hemodynamic changes that develop or persist post-procedure are required to better understand long-term outcomes and their prognostic implications.

TRISCEND II (Edwards EVOQUE Transcatheter Tricuspid Valve Replacement: Pivotal Clinical Investigation of Safety and Clinical Efficacy Using a Novel Device; NCT04482062) is a prospective, multicenter randomized controlled study comparing TTVR with OMT to OMT alone. Currently underway with a planned enrollment of 775 patients who have severe or greater functional or degenerative TR, it will evaluate the safety and long-term efficacy of the EVOQUE system up to five years.

#### 4.1.2 Intrepid Valve

The dual-stented, self-expanding Intrepid valve (Medtronic, Minneapolis, MN, USA), available in three sizes for the outer stent (43, 46, and 50 mm) with a 27 mm inner stent diameter is currently recruiting in the US for an early feasibility study (NCT04433065) evaluating device success and safety. Previously, the Intrepid valve achieved Food and Drug Administration (FDA) breakthrough device status after being deployed successfully via a transfemoral approach in three patients with severe TR as a compassionate use measure [72].

#### 4.1.3 TriSol Valve

The TriSol (TriSol Medical Ltd, Yokne’am Illit, Israel) valve consists of a self-expanding nitinol elastic frame that anchors to the TV annulus using axial forces and allows a secure fit without disrupting the anatomy of the native valve. The use of axial forces to anchor potentially reduces the risk of conduction system disturbance [73]. RV afterload mismatch is a potential complication of TTVR. Especially in the presence of underlying RV dysfunction, an increase in RV volume by eliminating TR can acutely worsen RV systolic function and subsequently increase afterload. The TriSol valve’s two leaflets close to form a dome shaped structure during systole which increases RV volume capacity by 20 mL and helps lower the acute increase in RV afterload [5]. As safety and procedural feasibility in animal studies have been demonstrated, a prospective, multi-center, first in-human early feasibility study (NCT04905017) of TriSol valve is underway and expected to provide insight into its safety and efficacy for moderate or worse TR.

#### 4.1.4 LuX Valve

The LuX valve system (Jenscare Biotechnology, Ningbo, China) is a radial-force independent orthotopic valve that is inserted through a transatrial approach after a minimally invasive thoracotomy. Unlike other valves, it secures fit by anchoring to the interventricular septum and to the native valve via two anterior clampers, responsible for the radial-force independent design. However, this carries a theoretical risk of injury to the septum and interventricular communication. To accommodate large annular diameters, it is available in 50-, 60- or 70-mm sizes. To date, preliminary small studies, mostly from China, have evaluated the feasibility of LuX system. Short term outcome assessed by a prospective observational study from China evaluating device success (defined as successful implantation and prosthetic valve function without major complications or device related mortality at 30 days) and safety of this system found it to be feasible in 11 out of 12 patients with severe to torrential TR. At 30 days, a significant reduction in TR (reduction ≥2 grades) on TTE and improvement in the NYHA functional class were reported [74]. One patient experienced device-related death on post-operative day 18 [74]. 12-month outcomes reported by another study confirmed persistent significant reduction in TR severity and improvements in quality of life at one year. However, one of the six patients who had a paravalvular leak died at the 3-month follow-up [75]. The TRAVEL (Transcatheter Right Atrial-ventricular Valve rEplacement With LuX-Valve) trial (NCT04436653) is currently recruiting in multiple centers in China and is expected to provide data on long term mortality and adverse events.

#### 4.1.5 SAPIEN 3 Transcatheter Heart Valve (valve-in-valve)

The Edwards SAPIEN 3 Transcatheter Heart Valve System (Edwards Lifesciences, Irvine, CA, USA) is well-established in the management of aortic stenosis. It recently received FDA approval for transcatheter replacement of pulmonary valve for pulmonary regurgitation and has extended its purview to the tricuspid valve, wherein there have been reports of successful valve-in-valve implantation of the SAPIEN 3 in the tricuspid position as compassionate use for patients who lack alternatives [76, 77].

#### 4.1.6 GATE System 

The NaviGate transcatheter heart valve (NaviGate Cardiac Structures Inc., Lake Forest, CA, USA) contains a trileaflet equine pericardial valve in a sutureless nitinol self-expanding stent [46]. It is built with ventricular graspers to facilitate anchoring and 12 atrial winglets with woven microfibers to help prevent injury to the compression system [44]. Available in four sizes ranging 36 to 52 mm, the NaviGate system has demonstrated early feasibility with excellent technical success in multiple reports, leading to compassionate use of the device in patients with severe symptomatic TR who are at high surgical risk. In a case series of 30 patients who underwent NaviGATE implantation on a compassionate use basis, technical success was achieved in 87% of the cohort and in-hospital mortality was 10% [78]. 100% of those who received the device had reduction in TR of ≥1 grade and 76% had mild or less TR at discharge. On follow-up, continued improvement in TR grade was observed between discharge and 30 days in 79% of the patients [78]. The investigators concluded significant reductions in TR severity and corresponding improvements in functional status with an acceptable in-hospital mortality in patients with severe, symptomatic functional TR [78].

#### 4.1.7 Other Valves

Other orthotopic transcatheter valve that have been used are the Cardiovalve (Boston Medical, Shrewsbury, MA, USA), and TriCares (TRiCares SAS, Paris, France). TriValve which is the largest registry worldwide for tricuspid valve interventions is expected to provide insight into real-world outcomes of TTVR and its incorporation into routine clinical practice. Outside of randomized controlled trials and implantation of available prostheses by highly experienced and skilled operators, real-world data incorporating inter-operator variability and heterogeneity of patient populations and operative practices are essential to evaluate outcomes on a global scale.

### 4.2 Heterotopic Valves

The rationale of implanting heterotopic caval valves is such that the anatomy of the native TV apparatus may not be suitable for prosthetic implantation despite the versatility of available devices and the various sizes [46]. Caval valves may also be used in cases where implantation of an orthotopic valve would not provide clinical benefit, such as in the presence of long-standing severe RV dysfunction and/or pulmonary hypertension that are beyond the stage of reversal. By preventing regurgitation of blood further down the inferior vena cava, the caval valve palliates symptoms of right heart failure such as hepatic venous congestion, ascites, subsequent right upper quadrant pain or abdominal discomfort, and pedal edema [79]. However, the inherent mechanism by which these valves work and their location precludes any improvement in RV hemodynamics and therefore, the implantation is primarily undertaken to palliate symptoms [46].

#### 4.2.1 TricValve

The TricValve (P+F Products, Vienna, Austria) is a bicaval valve system built to reduce caval reflux in both superior and inferior vena cava and abate systemic symptoms of right heart failure. The superior vena cava valve, available in 25- and 29-mm sizes is made of a long bovine pericardium skirt to curtail paravalvular leak and is housed within a nitinol frame. The IVC counterpart, available as a 31 mm or a 35 mm nitinol-based valve is designed with a short bovine pericardium skirt to prevent hepatic vein occlusion. Caval fixation relies on stent design, radial force and the extent of oversizing at the time of implantation [79]. The TricValve received CE mark approval in May 2021 and is also the only caval valve implantation device to receive CE mark approval till date. Previously it achieved FDA breakthrough device status. Currently there are two ongoing trials evaluating Tric Valve: the TRICUS STUDY (Safety and Efficacy of the TricValve Transcatheter Bicaval Valves System in the Superior and Inferior Vena Cava in Patients With Severe Tricuspid Regurgitation; NCT03723239) which a monocentric early feasibility first-in-human study, and the TRICUS STUDY Euro (NCT04141137), a multicentric pivotal trial geared at evaluating major adverse events at 30 days and improvements in quality of life at three months in about 35 patients.

#### 4.2.2 Trillium

Innoventric’s Trillium Stent Graft system (Innoventric, Ness-Ziona, Israel) consists of a bare metal stent with a sealing skirt to secure a tight fit in the IVC without occluding hepatic veins. It consists of multiple covered fenestrations that are arranged circumferentially in the right atrium. These fenestrations allow venous return into the right atrium and reduce venous pressure by controlling regurgitant flow from the TV [79]. The cross-caval stent graft is delivered with a 24 Fr delivery system via transfemoral venous access under fluoroscopic guidance. Multiple circumferential valves facilitate ease of device positioning even in the presences of pacemaker or ICD leads [80]. Endorsed to be a 10-minute skin to skin procedure, a multi-center, first-in-human study evaluating safety and prosthetic performance is underway (NCT04289870).

#### 4.2.3 Tricento 

Tricento (New Valve Technology, Muri, Switzerland) is a self-expanding bio-prosthetic valve made of Nitinol support structures and porcine pericardium. It consists of a 13.5 cm covered stent with landing zones in the superior and inferior vena cavae. It also consists of a short non-covered segment for hepatic vein inflow. Secure fit is achieved by oversizing in the area where the stent and caval veins overlap. The device can be customized to a maximum size of 48 mm and is delivered with the help of a 24 Fr delivery system transfemorally. Previously, results from first-in-human experience were made available [60]. Since September 2021, the recently registered TRICAR (Investigation of a Transcatheter Tricuspid Valved Stent Graft in Patients with Carcinoid Disease; NCT05064514) trial will be evaluating TRICENTO in 15 patients with carcinoid heart disease who are not candidates for surgery, for reduction in TR and improvement in quality of life.

## 5. Conclusions

The widespread prevalence of tricuspid regurgitation and the lack of effective, yet safe surgical options that can serve all patients have paved the path for innovative transcatheter interventions. Although TTVR is in its incipient stage, it is evolving at an exponential pace in response to incoming data from in-human experiences around the world. The last few years have been especially promising as these devices in the hands of experienced operators have continued to excel and provide results with evident and reproducible clinical benefit. Most importantly, TTVR has made its mark in the treatment of severe symptomatic tricuspid regurgitation, one that will only increase in importance with an aging population. It is important to acknowledge and appreciate the novelty of this approach, the indisputable lack of long-term data on safety, efficacy, morbidity, and mortality, and use the lessons learned from real-world experiences to provide a definitive and reproducible solution for patients with symptomatic TR.
